# Expanding the Manufacturing Approaches for Gastroretentive Drug Delivery Systems with 3D Printing Technology

**DOI:** 10.3390/pharmaceutics16060790

**Published:** 2024-06-11

**Authors:** Imola-Rebeka Turac, Alina Porfire, Sonia Iurian, Andrea Gabriela Crișan, Tibor Casian, Rareș Iovanov, Ioan Tomuță

**Affiliations:** Department of Pharmaceutical Technology and Biopharmacy, Faculty of Pharmacy, University of Medicine and Pharmacy “Iuliu Hatieganu”, 400012 Cluj-Napoca, Romania; imola.rebe.turac@elearn.umfcluj.ro (I.-R.T.); sonia.iurian@umfcluj.ro (S.I.); crisan.andrea.gabriela@elearn.umfcluj.ro (A.G.C.); casian.tibor@umfcluj.ro (T.C.); riovanov@umfcluj.ro (R.I.); tomutaioan@umfcluj.ro (I.T.)

**Keywords:** gastroretentive, drug delivery systems, floating systems, bioadhesive systems, swelling systems, 3D printing

## Abstract

Gastroretentive drug delivery systems (GRDDSs) have gained substantial attention in the last 20 years due to their ability to retain the drug in the stomach for an extended time, thus promoting an extended release and high bioavailability for a broad range of active pharmaceutical ingredients (APIs) that are pH-sensitive and/or have a narrow absorption window. The currently existing GRDDSs include floating, expanding, mucoadhesive, magnetic, raft-forming, ion-exchanging, and high-density systems. Although there are seven types of systems, the main focus is on floating, expanding, and mucoadhesive systems produced by various techniques, 3D printing being one of the most revolutionary and currently studied ones. This review assesses the newest production technologies and briefly describes the in vitro and in vivo evaluation methods, with the aim of providing a better overall understanding of GRDDSs as a novel emerging strategy for targeted drug delivery.

## 1. Introduction

Oral dosage forms have gained significant importance for human administration as simple, non-invasive medications that provide advantages in terms of flexibility in formulation, facile storage, and transport or ease of handling. In addition, oral dosage forms are associated with high patient compliance and, therefore, they are among the most prescribed dosage forms [1,2,3]. Although conventional oral dosage forms are highly used, they present some disadvantages. First, their short gastric retention times (GRTs), coupled with the low surface area for drug absorption in the stomach, have a negative impact on the bioavailability of drugs that require an acidic pH for absorption. Second, when a local effect is desired, a limited GRT impairs the achievement of the sought-after therapeutic effects [4]. These are among the primary factors that highlight the importance of gastroretentive drug delivery systems (GRDDSs).

GRDDSs play an important role in the delivery of APIs in the upper gastrointestinal tract to overcome a limited GRT. By prolonging the GRT, substances that are highly soluble in the acidic environment will have a better solubility and thus, a higher concentration in the gastric fluid, which will promote absorption. Additionally, the therapeutic activity for short half-life medicines is prolonged by maintaining an effective drug concentration in the systemic circulation for an extended period of time [5]. Patient compliance is another benefit gained through the reduction of dose frequency as a result of the sustained and controlled drug release in the stomach [6]. GRDDSs have shown benefits not only for systemically absorbed drugs but also for the local treatment of gastric or duodenal ulcers and esophagitis. Due to their prolonged residence time in the stomach, they can eradicate *Helicobacter pylori* from the submucosal tissues of the stomach [5].

To develop GRDDSs, several approaches to increase the GRT have been applied: the modulation of product density, such as to be either denser or less dense than the gastric liquid, to resist the peristaltic movement by settling at the bottom of the stomach or floating on the surface of the gastric fluid, respectively; the blockage of the pyloric sphincter using “plug-type” swelling or expanding systems; the application of an external magnetic field to increase the retention of magnetically active systems; and increasing the interaction with the stomach wall by imparting mucoadhesion [5]. Although many GRDDSs have been developed, floating, swelling, and mucoadhesive dosage forms are the most studied ones.

Regarding the fabrication of GRDDSs, the majority are manufactured using conventional technologies applied to oral dosage forms, with direct compression and wet granulation being the most popular. However, in the last few years, 3D printing technology has emerged as a suitable method to obtain a variety of dosage forms, ranging from simple to highly complex shapes. Through the proper selection of excipients, coupled with the design flexibility provided by 3D printing, the facile preparation of modified-release dosage forms, including GRDDS, is feasible [7]. This technology is being explored due to the advantages it brings compared to the traditional ones, namely, simple manufacturing steps, flexible dose adjustment, flexible geometry and internal structure of the dosage forms, and its suitability for the preparation of polypills [7,8]. Among the two dozen existing 3D printing technologies, fewer than ten are adaptable to the processes and materials used in pharmaceutical applications [9]. Of those suitable for pharmaceutical manufacturing, two have been applied for the manufacture of GRDDSs so far, i.e., fused deposition modeling (FDM) and semi-solid extrusion (SSE), also known as pressure-assisted microsyringe extrusion (PAM). Both are extrusion-based technologies, relying on the layer-by-layer deposition of molten mixtures (in FDM) or semi-solid materials (in PAM) on a Build Platform to obtain the desired structure, which is defined using a specialized software [8,10]. Although FDM is very popular in pharmaceutical applications, its main disadvantage is that it requires high-temperature processing, making it unsuitable for thermolabile drugs, while SSE has the advantage of printing at low temperatures [10].

The objective of this review article is to summarize the existing information about GRDDSs, including the recent advances in perfecting their formulation and fabrication to achieve a prolonged GRT and high bioavailability of the APIs, with a special focus on 3D printing as a promising technology for the manufacturing of these products. Moreover, a review of in vitro and in vivo evaluation methods was also performed in order to give an overview of the methods used in the analysis of GRDDSs.

## 2. Physiology of the Stomach and Factors Affecting Gastroretention

Knowing the key anatomical and physiological information about the stomach as well as details about gastric emptying rate is crucial for the successful formulation of GRDDSs. The fundus, body (proximal and distal corpus), and antrum are the three main sections of the stomach. The fundus is a muscular bag that relaxes upon food intake to promote gastric accommodation and stores the gas that is produced during digestion [11,12]. The corpus acts as a reservoir, which accommodates incoming food until the chymus (fluidized mixture consisting of ingested food particles ground by the digestive juice) from the antrum is propelled towards the pyloric sphincter [13,14,15]. The antrum is the principal site for grinding and functions as a gastric pump that propagates the chymus to the pylorus via peristaltic waves [11,12]. The pyloric sphincter is responsible for the flow of the chymus in the duodenum through peristaltic movements. While the average volume of an empty human stomach is about 25–50 mL, the volume increases to 1–1.5 L after an average meal. Gastric pH in the fasted state varies between 1 and 3; it rises up to 5.5–7 during the ingestion of the meal, decreases to 3–4 when it is half-empty, and returns to the basal value after the stomach is fully empty [12]. The stomach emptying rate varies depending on the type and components of food ingested, while liquids and solids can have different emptying mechanisms [11]. Gastric motility is defined as the sum of contractions and relaxations of smooth muscles of the stomach’s wall, which participate in gastric accommodation, mixing, emptying, and migrating motor complexes. Gastrointestinal (GI) motility in the fasted state consists of four phases, and it is called the migrating motor complex. In the fasted state, a sequence of contractions occurs every 120–180 min, which changes in the fed state, which is divided into four phases [6,15]. Phase I is a quiescent period with rare contractions (basal phase), while phase II consists of irregular, low-amplitude, and intermittent contractions that last from 20 to 40 min; in this phase, bile excretion and mucus discharge also occur (pre-burst phase). In phase III, a short burst of irregular high-amplitude contractions was observed (10–20 min of duration), occurring every 90–120 min [6,16]. In this phase, the frequency of regular contractions is maximal, which causes the material to migrate distally [5]. Finally, phase IV represents the transition to period I, which lasts between 0 and 5 min [6,16,17].

The most important factors affecting gastric retention are fed/unfed states, the nature and caloric content of the meal, feeding frequency, sex, and age. Gastric emptying is much more rapid in the fasted state. Thus, some GRDDSs require the presence of food and sufficient liquid to help the buoyancy of dosage forms and prolong their gastric residence time [5,15,18]. If the dosage form is administered when peristalsis starts, the gastric retention rate will be short, whereas after meals, peristalsis will be delayed and can increase the retention time of GRDDSs [19]. A high-calorie and nutritious meal full of proteins, healthy fats, and fibers slows gastric emptying; in other words, it increases gastric retention time. The gastric retention time increases after the ingestion of multiple meals per day, whereas a single meal per day decreases gastric retention. Females and geriatric patients have a slower gastric emptying time than men and neonates [6,19].

## 3. GRDDS Classification

**Figure 1:**
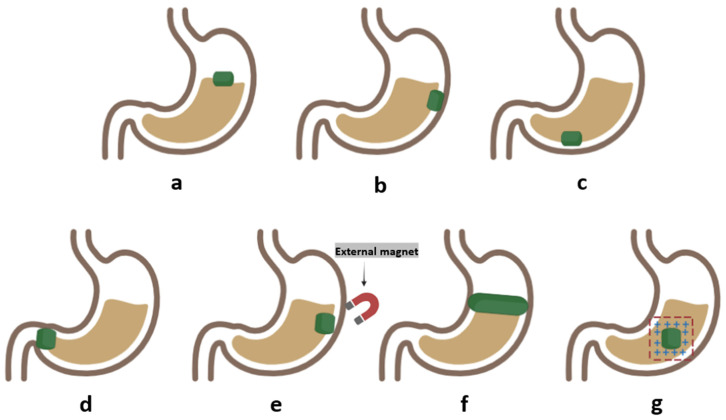
A brief illustration of the GRDDS classification: (**a**) floating systems, (**b**) mucoadhesive systems, (**c**) high-density systems, (**d**) expandable systems, (**e**) magnetic systems, (**f**) raft-forming systems magnetic systems, and (**g**) ion-exchange resin systems.

GRDDSs include floating, expandable, bio/mucoadhesive, raft-forming, magnetic, ion-exchange, and high-density systems, as shown in Figure 1 [1]. Although currently there are seven different approaches to developing a GRDDS (as shown in Figure 1), extensive studies have only been conducted on floating, bioadhesive, and swelling systems [20]. These systems can be classified into single or multiple-unit systems, depending on the involved preparation techniques and the types of dosage forms in which they are formulated, as shown in Table 1 [21]. For the development of GRDDSs, various materials, like resins, mucoadhesives, low-density materials, raft-forming, magnetic substances, and superporous hydrogels, need to be used [6,22].

### 3.1. Floating Drug Delivery Systems (FDDSs)

Floating systems are based on the principle of buoyancy in the GI fluid, having a density less than the density of the GI fluid (1.004–1.010 g/mL), which allows a continuous drug release in the stomach [2,5,6,45]. The polymer type, viscosity grade, presence of a wicking agent, and swelling enhancers influence the rate of swelling of the polymer from the dosage form, which influences the buoyancy of the floating dosage form and the in vitro drug release rate [5,46]. Floating systems may be inherent low-density systems, or they may achieve their low density upon contact with gastric fluid [47]. Flotation can be achieved by the incorporation of low-density polymers with swellable properties or by the generation and entrapment of gas using carbonates in the formulation [45]. Based on this criterion, they can be classified into non-effervescent and effervescent floating dosage forms. Optimal floating tablets must feature two characteristics: high porosity to promote flotation on the surface of gastric chyme as well as sufficient hardness to withstand destruction due to the peristaltic waves generated by the stomach [45]. FDDSs are desirable for drugs with optimal absorption at a lower pH, being useful both locally in GI infection eradications, such as *H. pylori* eradication or inflammatory diseases such as ulcers, and for the systemic action of APIs with a low bioavailability and absorption rate in the small intestine [48].

Similar to other GRDDSs, there can be single- or multiple-unit FDDSs. Single-unit systems showed a high variability of gastric emptying time due to their all-or-nothing emptying processes [49]. Multiple-unit systems, however, may be a more attractive choice, as they showed less inter- and intra-subject variabilities in drug absorption and lowered the possibility of dose-dumping [50].

#### 3.1.1. Non-Effervescent Floating Systems

This class of floating systems can achieve flotation by the the incorporation of polymers that gel and swell (such as hydroxypropyl methylcellulose (HPMC), polyacrylate, and sodium alginate) or by foaming. When these polymers reach the gastric fluid, they swell due to hydration and form a gel layer with entrapped air around the core; thus, the drug release is slow and gradual [2]. Hydrodynamically balanced systems belong to the same category as microspheres and microporous systems containing a matrix-forming polymer and gel-forming hydrocolloids. Upon contact with the gastric fluid, hydrocolloids hydrate and form a gel barrier around the polymer–API mixture, thus prolonging the GRT of the dosage form and ensuring sustained drug release as well [51]. Ali et al. formulated a hydrodynamically balanced drug delivery system with ofloxacin using capsules made of HPMC and polyethylene oxide (PEO WSR 60K). Cellulose acetate phthalate, liquid paraffin, and ethyl cellulose were used as release modifiers to maintain the release of the drug over a period of 12 h [52]. The most commonly used methods for developing floating tablets are direct compression and the combination of FDM and HME [53,54]. Souza et al. developed a floating tablet consisting of sildenafil citrate and HPMC K100 CR HPMC K4M (9:2) by direct compression, and almost all the tablets had a low floating lag time, with a first-order kinetic dissolution and a total in vitro floating time of more than 24 h [55]. Eberle et al. studied the drug release and flotation mechanisms of a GRDDS formulated with functionalized calcium carbonate, which promised a good flotation behavior due to the low density (approx. 0.6 g/cm^3^) and high specific surface (approx. 70 m^2^) while displaying sufficient hardness of the resulting compacts. The results revealed that functionalized calcium carbonate is a promising pharmaceutical excipient as the prepared floating tablets showed no floating lag time in vitro and in silico, which lowers the risk of possible premature gastric emptying. Despite having a porosity of 60%, the formulations had a high resistance given that they were able to withstand peristaltic waves in the stomach [45].

Double- or multiple-layer FDDSs are currently being studied as promising approaches to combine multiple APIs into one formulation or to include immediate-release and extended-release layers into one formulation to rapidly ensure an effective concentration in the organism and maintain it for a determined period. He et al. created bilayer floating tablets consisting of two layers. The first layer consisted of pioglitazone, a polymer and disintegrating agent, for rapid disintegration and effectiveness, while HPMC was used as a sustained-release polymer in the metformin layer. The fabrication was carried out through the single-compression method. A complete release of pioglitazone was achieved in 5 min. Metformin was released within 12 h via diffusion, resulting in a steady metformin concentration in the blood. Thus, this bilayer formulation proved to be a possible solution in the management of diabetes mellitus [24]. Ullah et al. prepared a bilayer floating tablet by the direct-compression method with clarithromycin and pantoprazole for the treatment of *H. pylori*. In the formulation, various hydrophilic polymers were used, such as chitosan, HPMC, and sodium alginate, in a ratio of 1:1:1. The pantoprazole layer had 95% of drug release in 2 h, whereas clarithromycin demonstrated a sustained release for up to 24 h. Although in vitro studies proved to be efficient, further evidence is required to demonstrate a sustained release in vivo [56].

Another interesting technique to develop FDDSs is the preparation of oral in situ gelling liquid formulations to achieve a prolonged floating time and gradual drug release rate. The polymeric API mixture is formulated in solution form and undergoes a gelation process once it is administered. Currently, used polymers for the in situ gel formation are gellan gum, alginic acid or sodium alginate, pectin, chitosan, and poly-caprolactone, but among all, sodium alginate is preferred [57]. Fungfoung et al. formulated an oral hydroxycitric acid (HCA) containing in situ gelling liquid, combining sodium alginate as a gelling polymer, with calcium carbonate as a cross-linker, and HPMC K100. The formulation presented a rapid gelation time of less than a minute in a solution containing 0.1 N of HCl, gradually released the API within 8 h, and had a floating time of more than 24 h [29]. Similarly, Sharma et al. created an oral spiramycin-containing in situ gelling formulation with HPMC and sodium alginate. A total floating time of more than 12 h was observed, and the formulation released between 80–100% of spiramycin within 12 h, thus proving the sustained release of APIs [30].

Multiple-unit systems can include hollow microspheres, known as microballoons, first developed by Kawashima et al. in 1992, and consist of a hollow center and external polymer layer in which the drug is loaded. These microballoons can be achieved by solvent evaporation or emulsion–solvent diffusion methods [21]. They can be developed using various polymers, such as Eudragit S, calcium alginate, polycarbonate, pectin derivates, or agar. The floating behavior and drug release will highly depend on the ratio and amount of plasticizer, polymer, and solvent [22]. Uthumansha et al. created telmisartan-loaded alginate beads by the emulsion–gelation method using calcium chloride (CaCl2) as a cross-linking agent and evaluated their entrapment efficiency, in vitro buoyancy, in vitro drug release, and in vivo efficacy in an induced animal model. The objective of the study, which was to enhance the oral bioavailability of telmisartan by creating oil-entrapped telmisartan beads, was successfully proved by an in vivo study that confirmed an extended anti-hypertensive activity of the API. In vitro studies confirmed the in vivo results as the formulation proved to have a high entrapment efficiency percentage and remained buoyant for 7 h while showing an extended release over 12 h in a simulated gastric fluid [58]. Microporous systems are another category of multiple-unit FDDSs, obtained through the encapsulation of a drug reservoir inside a microporous compartment, presenting pores along the walls. The microporous compartment, upon contact with the gastric fluid, allows the diffusion of APIs at a slow and controlled rate [51].

Preparing stable floating formulas by foaming is a newly emerging technique that has promising results and needs further investigation. Foaming systems are prepared by melting the matrix, dispersing the API in the melted polymer (e.g., polyethylene glycol 4000), and foaming the mixture with different gases using an ultrasonic homogenizer. After foaming, the hot dispersion was molded into a steel mold to create the dosage forms. Using scanning electron microscopy (SEM), the foam particles were successfully analyzed, observing acyclovir crystals in the matrix. In ex vivo studies, the samples showed mucoadhesive properties on the surface of the rat stomach, which is a promising result that deserves further investigation [33]. Another study confirmed that foaming FDDS capsules containing verapamil hydrochloride showed a prolonged release of APIs in vivo, and gastroscopic images revealed the gastric retention of foaming capsules in the stomach [31].

#### 3.1.2. Effervescent Floating Systems

There are two types of effervescent systems: gas-generating and volatile liquid-containing systems, among which, gas-generating systems are the most evaluated. Gas-generating systems, as their name suggests, are characterized by gas production as a result of the reaction of incorporated bicarbonates/carbonates on one side and gastric fluid or coformulated acids on the other. The generated gas is entrapped in the hydrocolloid gel matrix that the polymers form and ensures flotation during drug release [21]. Sodium bicarbonate is mostly used as an effervescent agent, while organic acids, like citric or tartaric acids, are often added as they speed up the gas generation process and contribute to a longer buoyancy by reducing the density [5,6]. An ideal coating material for effervescent floating systems should be used for maintaining a long-term drug dissolution and should meet a few criteria in order to give good results: it should have high water permeability to be able to initiate the effervescent reaction and thus, the floating process; wet or hydrated coatings, however, should not be permeable for the generated CO_2_ to maintain flotation; and it should be flexible enough in a hydrated state to be able to resist the pressure of the resulting gas to prevent rupture [23,59]. A study conducted by Chen et al. focused on the development of losartan-containing swellable and floatable GRDDSs based on a combination of sodium carboxymethylcellulose (NaCMC-450 cps) and hydroxyethylcellulose (HEC 250 HHX). Sodium bicarbonate was employed as a gas-generating agent. The influence of formulation factors on the bioavailability of the drug and on the extent of active metabolite formation was evaluated. Different compression pressures, ranging from 0.25 to 1 ton-force, were employed for the fabrication process. The influence of the applied compression pressures on the in vitro swelling and floating capacity of the products was also evaluated. The results revealed that tablet formulations containing a ratio of HEC and NaCMC greater than 60:40 compressed at 0.5–1.0 tons were able to float immediately or after a short lag time, with a total floating time of more than 8 h. Additionally, the tablets with a floating time of over 24 h were able to increase the bioavailability of losartan to over 160% relative to its bioavailability from the immediate-release product [4]. Amrutkar et al. created multiple-unit effervescent floating tablets using Eudragit^®^NE 30D-coated zolpidem tartrate floating pellets and observed that 70–90% of the pellets were floating up to 10 h in 0.1 N of HCl as a dissolution medium, with a floating lag time (FLT) of 5–15 min. The floating time of the tablets increased proportionally with the polymer content of the pellets and the coating. However, increasing the amount of the effervescent agent reduced the floating time as it caused faster and higher CO_2_ formation, which was attributed to an increase in polymer concentration and a decrease in pellet density [23].

### 3.2. Expandable Systems

An expandable system achieves a prolonged gastric residence time by increasing its volume or unfolding upon contact with the gastric fluid and minimizing its dimensions after drug release for further elimination from the GI tract [41]. These systems need to meet three major criteria: first, they must have the proper dimensions that allow them to be easily swallowed and for adequate patient compliance; second, the swollen size of the dosage form after reaching the gastric fluid should be greater than the pyloric sphincter’s diameter to prevent their expulsion from the stomach via peristaltic waves; and third, they must have high rigidity in order to withstand the mechanical contractility of the stomach [21,41]. Thus, there is a need for a polymer or a combination of polymers with an appropriate molecular weight, a viscosity grade, and good swelling properties in order to reach a prolonged GRT [5]. A major quality of swelling systems is that they do not depend upon the fed or unfed state, unlike floatable systems [4]. The formulation of expandable systems relies on two strategies, either the swelling properties and or the unfolding properties of the system [21]. Unfoldable systems are prepared from biodegradable polymers and APIs in a large size and further folded and encapsulated into a pharmaceutical carrier, like a gelatin capsule [21,41]. For the formulation of swellable systems, there is a need for hydrophilic polymers, like HPMC, polyethylene oxide or carbopol, xanthan gum, pectins, gellan gum, or alginate, which are capable of absorbing water from the gastric fluid, resulting in polymer swelling and plasticization, an increase in diffusion coefficient, and finally, polymer erosion, which determines the release of the API [21,60]. In a preliminary study conducted by Chen et al., it was found that hydrocolloid tablets made of polyethylene oxide (PEO 8.000K) had the largest swelling index, followed by HEC, and sodium carboxymethyl cellulose had the lowest swelling index [4]. Polymers should have a slow water absorption rate, which allows a prolonged API release [61]. Smart hydrogels, like N-isoproylacrylamide (PINPA), were analyzed in a study by Fu et al. (2010) and were shown to change their swelling properties according to the pH, temperature, and solvent composition. Although PINPA showed different swelling properties through in vitro release experiments, there is a further need to investigate their in vivo performance [62]. Ahmed et al. studied the swelling properties of tablets made with itopride hydrochloride and different types and amounts of swelling polymers, like gellan or xanthan gum, sodium alginate, and pectin. The results showed that xanthan gum was the most favorable polymer, exhibited a considerable expansion rate, and maintained its swollen form for 24 h while showing a sustained API release behavior. In vivo tests using X-ray showed that the tablet’s GRT in the fed state was 6 h, which proved that expandable system formulations can achieve a sustained release [40]. Similarly, El-Zahaby et al. prepared a swelling system of levofloxacin hemihydrate with the above-mentioned polymers and studied the effects of adding cross-linkers, like calcium and aluminum chloride, on the drug release. The addition of cross-linkers to gellan gum decreased the drug release rate but increased the release rate for the alginate-, xanthan gum-, and pectin-based formulations. However, formulations with no cross-linkers showed the longest sustained release, which demonstrates that the addition of cross-linkers does not promote a sustained release [63]. Although they seem to provide successful results, a major drawback of these systems can be their possible accumulation in the stomach, which can also lead to serious implications for the patient; so, a fast biodegradation process of the excipients is needed after drug release to prevent further complications [23,41].

Superporous hydrogels have the same principle as expandable systems, but they consist of a superporous cross-linked hydrophilic polymer, which is characterized by a rapid swelling ratio of 1:100 or more, much higher and quicker than polymers in swelling systems [5,64]. The polymers used are either superabsorbant polymers (SAPs) or superporous hydrogels (SPHs). They both consist of acrylamide or salts of acrylic acid, but the method of synthesis varies, e.g., in the case of SAPs, the synthesis method is through inverse suspension, while in the case of SPHs, it is mostly through an aqueous solution. The difference between the two types of polymers is the swelling rate as the swelling kinetics of SAPs depends on the size of the product, and for SPHs, swelling kinetics is always fast, regardless of the final product size [65]. In addition to a cross-linked polymer, a superporous hydrogel system also contains the following ingredients: initiator, cross-linkers, foam stabilizers, and foaming aids or agents [6]. When superporous hydrogels encounter the water from the gastric fluid, a phenomenon called capillary wetting occurs through the system’s large open pores (as a result of swelling), which increases the contact surface between the dosage form and the gastric fluid; thus, better drug bioavailability will be ensured [5,6,64]. As a result of the rapid swelling property of the superporous polymer, peristaltic waves are incapable of forcing the dosage form through the pyloric sphincter; thus, a prolonged gastric retention of the system is ensured. A major limitation is that superporous systems are mechanically very fragile, which, in many cases, can lead to the destruction of the dosage form by peristaltic waves. However, this problem can be solved by regulating their mechanical strength [21]. Hydrogels are classified into three categories based on their swelling and mechanical properties. The first category is called conventional superporous hydrogels, characterized by a high swelling ratio, mechanical fragility, and quick swelling. The second category also includes quick swelling but a moderate swelling ratio and superior mechanical fragility, while the third category includes hybrid superporous hydrogels with a high mechanical strength, which has demonstrated promising results regarding future use in GRDDSs [21,65]. A novel semi-IPN (interpenetrating polymer network) was prepared by Grosso et al. for future use as matrices for GRDDSs, using guar gum and a synthetic copolymer. Novel synthesized polymers were loaded with amoxicillin for the treatment of *H. pylori* infections. Drug-loaded semi-IPNs demonstrated a controlled and sustained release of amoxicillin, and the matrix proved to be a well-structured, mucoadhesive, and superporous system that can be used in further dosage forms [66].

### 3.3. Bioadhesive/Mucoadhesive Systems

These systems present bioadhesive properties, having the capacity to adhere to the stomach wall after ingestion, and remain attached for a longer time, despite gastrointestinal motility. Based on the properties of the polymer, these systems can be cytoadhesive when they adhere to the epithelial surface and mucoadhesive when they bind to the mucus layer, interacting with mucin by electrostatic interactions, hydrogen bonding, hydrophobic bonding, and disulphide bond formation [21,22]. These systems represent a great option to treat local infections. Their formulation includes natural polymers, like sodium alginate, pectin, gelatin, and guar gum, and semi-synthetic polymers, such as chitosan, carbopol, lectins, polycarbophil, carboxymethylcellulose, and gliadin [5,6]. The adhesion is mediated by hydration, bonding, or receptor interactions [2,6]. Ideally, the polymers used in this formulation should be inert, non-irritating, and non-toxic, with good adherance properties. Characteristics like the shape, flexibility, density of cross-linking, ability to form H-bonds, hydration behavior, and charge of the polymer have a great influence on the contact strength and mucoadhesive characteristics [22]. Due to adhesion, there is constant contact between the drug and the local microenvironment, which is a great advantage, especially when used in local treatment because it also requires a lower drug concentration. However, they also present some disadvantages as there is a possibility for the dosage form to adhere to the esophagus, resulting in possible lesions, and they also have a lower resistance to housekeeper waves and decreased bioadhesion during mucus layer renewal; so, they can be easily expulsed from the stomach [21]. So, mucoadhesive systems may cause potential drug-induced injuries, ranging from local irritation to perforation depending on the drug’s ulcerogenic properties [23]. Similar to floating GRDDSs, they can also be single-unit or multiple-unit dosage forms, and they can be formulated into tablets using the compression method or 3D printing [21,42]. Multiple-unit dosage forms, known as bioadhesive microspheres, are much more efficient and relevant since they combine the advantages of conventional microspheres with those of mucoadhesive systems. Microspheres can be made of bioadhesive polymers, or they can just be bed-coated with them [21]. Alginate–chitosan-based mucoadhesive microparticles containing puerarin were prepared by Hou et al. using the emulsification–internal gelation method. Chitosan and calcium were used as cationic components, and alginic acid was the anion, where alginate acted as the drug-loaded bead and chitosan served as a coating shell. In vitro studies revealed that puerarin mucoadhesive microparticles showed an extended gastric residence time, adhering to the gastric wall’s surface and improving the bioavailability of puerarin for local ulcer treatment. Thus, mucoadhesive systems can be a future strategy for local treatment in the stomach [43].

There is a newly emerging bioadhesive system that uses nanomaterials to formulate GRDDSs. Nanoparticles are materials with dimensions below 100 nm, currently used in modern medicine in various ways, such as X-ray, presently being studied in the pharmaceutical field as well [67]. Umaheshwary et al. formulated mucoadhesive gliadin nanoparticle-containing amoxicillin (AGNP) as an attempt to eradicate *H. pylori* and rhodamine isothiocyanate-entrapped GNP to evaluate in vivo mucoadhesion in albino rats. An initial amoxicillin burst release was observed due to the dissolution of amoxicillin crystals from the surface of nanoparticles. The results showed that with increasing gliadin concentration, the release rate of amoxicillin from AGNP decreased dramatically, contrary to the in vivo studies, which demonstrated that the mucoadhesion capacity of gliadin increased with its concentration. However, pepsin can increase the release rate if it is low. Regarding drug loading, the higher the drug concentration, the higher the drug release because, in the case of high drug loading, drug particles are close and have a better chance to come into contact with the GI fluid. Thus, GNP proved to be effective in the use of *H. pylori* eradication [68].

### 3.4. Magnetic Systems

Magnetic systems are based on the magnetic attraction between a dosage form with the incorporation of a magnetically active ingredient and an external magnet placed on the abdominal area near the stomach in a specific position. Due to the attraction between the magnetically active systems, the dosage form is retained in the stomach for a prolonged period of time [5,21]. Several studies conducted in the early 2000s showed a gradual drug release profile of acetaminophen [69] and acyclovir [70] from these types of systems, but due to their major setbacks, such as the need for external magnet use, these formulations showed low patient compliance and were not investigated further [5,21]. Fujimori et al. studied the effects and gastric residence time of magnetic systems with and without an external magnet using acetaminophen bilayer magnetic tablets prepared by alternating drug layers with magnetic layers containing ferrite. The study revealed that the area under the plasma concentration–time curve (AUC) of acetaminophen increased by about 2-fold when an external magnet was used. Moreover, the mean residence time of the tablets was prolonged when using the external magnet, which demonstrated that the influence of the external magnet has a great impact on the bioavailability of APIs and the gastric residence time [69]. Groning et al. also studied the influence of external magnets on a formulation made with acyclovir as the API and with integrated magnets, using the commercially available immediate-release acyclovir tablets as a reference. The results confirmed that the use of an extra corporal magnet placed on the stomach significantly increased the AUC, and the plasma concentrations of acyclovir were much higher after 7, 8, 10, and 12 h, which demonstrates the efficacy of magnetic systems in prolonging the GRT [70]. Hao et al. prepared a novel stomach-specific sinking magnetic microparticle-containing dosage form (SMMP) loaded with amoxicillin with a high density, which sank near the pyloric section in the stomach. The SMMP was approximately 5 μm in size, contained Fe_3_O_4_ nanoparticles, and had strong magnetism both in vivo and in vitro. These dosage forms sank in 120 s in vitro, and the settling time decreased to 20 s when using a magnetic field. In vivo data showed a gastric retention rate of 8 h, which could be prolonged by using an external magnet. Thus, the amoxicilin-loaded SMMP showed significantly greater eradication of *H. pylori* compared to traditional dosage forms [18,71].

### 3.5. Raft-Forming Systems

Raft-forming systems are designed to form a physical barrier on top of stomach content in the form of a floating gel barrier. They consist of an in situ gel-forming polymer, mainly sodium alginate, that also contains carbonates or bicarbonates as effervescent agents. Upon contact with the gastric fluid, sodium alginate forms a cohesive gel entrapped with CO_2_, wherein each proportion of the liquid swells, which explains the flotation of the dosage form, forming a continuous gel floating layer, called a raft [21,37,38]. Thus, these systems are beneficial for local gastroesophageal oral therapy, mainly with aluminum hydroxide or calcium carbonate for gastroesophageal reflux treatment, due to their raft-forming property, as well as for *H. pylori* eradication [5,72,73]. Manna et al. formulated an alginate and HPMC mix-based raft-forming system of tinidazole and evaluated the raft strength, raft volume, and floating lag time. For the formulation, a solid dispersion of tinidazole was prepared by the kneading method using methanol and PVP. The mixture was dried, then added to the alginate HPMC mixture, and compressed into tablets. The products with higher sodium alginate concentrations had the highest raft strength among all formulations. All the formulations presented a floating lag time between 40 and 60 s and a total floating time of 8 h. Thus, this study proved that there is a possibility of formulating anti-acidic raft-forming systems using a combination of HPMC, alginate, and PVP as matrix-forming agents, with a positive impact on the drug’s bioavailability [38]. Hampson et al. created alginate-based raft-forming tablets to provide anti-reflux/antacid symptom relief and analyzed the behavior of the different antacid systems that were formulated. They concluded that products with high acid-neutralizing capacities and no sources of calcium ions formed rafts with low strength, weight, and volume, appearing as floating precipitates. However, the products with low acid-neutralizing capacities with medium to large weight volumes and a source of calcium ions formed strong rafts, which turned out to be more resistant to acid reflux in vitro tests [37]. Wannasarit et al. developed a liquid raft-forming system comprising the solid dispersion of *Centella asiatica* extract and Eudragit^®^ EP (GR-SD) to improve the treatment of gastric ulcer. The dosage forms had an FLT of 30 s and released more than 80% of the active glycosides from the Centella extract in approximately 8 h. The in vivo tests on rats confirmed the efficacy of glycoside raft-forming systems on the healing process of indomethacin-induced gastric ulcer, which is a potential successful dosage form for future treatment [36]. Youssef et al. created an alginate- and gellan gum-based raft system of metronidazole for the treatment of *H. pylori*, developing formulations with different lipid ratios to prolong the GRT of metronidazole in the stomach. The formulations with alginates had a floating duration of less than 60 s, total floating time of more than 24 h, and sustained drug release with the required gelation capacity. Gellan gum achieved successful floating properties as well but had a low gelation capacity. The incorporation of lipids into the formulation showed an improved sustained release and formulation stability by forming a reversed micellar phase, making these dosage forms excellent candidates for *H. pylori* eradication [74].

### 3.6. Ion-Exchange Resin Systems

These systems contain a water-insoluble cross-linked polymer, called resin, which can be cationic or anionic, and an API. Since the gastric pH is acidic, for a prolonged gastric release of the API, a cationic polymer is needed, and only cationic APIs can be integrated into the dosage form. When the cationic polymer encounters an anionic polymer, drug ions will be absorbed into the resin matrix. When API-loaded resins reach the gastric fluid, protons will replace the cationic APIs, and thus, the API will be released into the gastric fluid. The release rate of the resins depends upon the particle size, cross-linking density, and ionogenic group type, as well as the nature of the drugs, ionic environment, and test solution. The most common resins used are polystyrene and polymethacrylate polymers [75]. Daihom et al. aimed to develop and evaluate a domperidon-loaded resinate complex with a controlled drug release to determine whether ion-exchange systems can be used for the further fabrication of gastroretentive formulations. The in vitro drug release revealed an incomplete release of the drug due to the entrapment of some molecules in the deep pores of the matrix, but the overall behavior of the system, showing a sustained drug release, proved that ion-exchange systems should be further investigated to achieve sustained-release formulations [76].

### 3.7. High-Density Systems

As their name suggests, these systems use their high density (weight) as a retention mechanism, which must exceed the gastric fluid’s density (1.004 g/mL) [6,77]. High-density systems can extend their gastric retention time from an average of 5.8 to 25 h [78]. Because they usually have a density of 2.5–3 g/mL, they instantly sink through the gastric fluid to the bottom of the stomach and are locked in the rugae of the stomach. As a result, they can withstand the in vivo peristaltic waves of the stomach and remain intact for a prolonged period, allowing the slow diffusion of APIs [5,19]. Barium sulfate, iron powder, titanium oxide, and zinc oxide were incorporated into the dosage form to increase the density. However, the major drawback was the increased size of the dosage form [1,5]. The information on high-density systems is currently limited; thus, further investigations are required to prove the efficacy of these systems [2].

### 3.8. Combinations

Although GRDDSs can have a great impact on drug bioavailability and patient compliance, some disadvantages do exist. Floating systems are highly dependent upon the fed state, bioadhesive systems can be affected by constant mucus turnover, and swelling systems suffer from the lack of integrity. Considering the disadvantages, there is a possibility of combining various systems to compensate for the disadvantages [1]. Nigusse et al. (2021) combined the three retention mechanisms to develop a GRDDS with ranitidine hydrochloride, an H_2_ receptor antagonist with a short biological half-life, low bioavailability, and narrow absorption window; these characteristics made it an ideal candidate for a GRDDS. In the study, polymers with bioadhesive and high swelling properties were used, mainly HPMC and sodium carboxymethylcellulose (NaCMC), hydroxypropylcellulose (HPC), and carbopol, as well as effervescent agents, mainly sodium bicarbonate. The formulations showed a floating duration of 12 h, an ex vivo mucoadhesion time of 12 h, and good swelling properties, with the matrix integrity being maintained for up to 12 h as well. They observed that the floating lag time decreased by increasing the amount of sodium bicarbonate because it was directly proportional to the amount of CO_2_ produced in the tablet. Thus, to achieve a minimal floating lag time, a high amount of sodium bicarbonate is needed. By increasing the amount of the polymer, an increase in bioadhesive strength was observed. Hence, an increase in the amount of the polymer was attributed to rapid swelling properties [20]. Another combined delivery system was prepared by Liang et al., consisting of a mixture containing Sangelose^®^ (hydrophobically modified HPMC) and HPMC polymers, incorporating ciprofloxacin hydrochloride as the API. Sangelose^®^ has a gel-forming capacity due to its high viscosity, and when combined with HPMC, it is able to float. Thus, this system relies on mucoadhesion and flotation to achieve an extended ciprofloxacin release to achieve an optimal antibiotic treatment. The results showed that the formulation was able to swell to a diameter above 11 mm and had a total floating time of more than 24 h. The formulation with the polymeric matrix consisting of Sangelose^®^ and HPMC K15 in a proportion of 50:50 (*w*/*w*) showed a sustained release of ciprofloxacin for 12 h, which proved that by combining multiple delivery systems, an improvement in the drug release rate and drug bioavailability can be achieved [79].

## 4. Three-Dimensional Printing as an Innovative Technology for GRDDS Fabrication

Three-dimensional printing represents one of the revolutionary methods for improving GRDDSs, allowing the production of complex drug delivery systems with customized drug release profiles for personalized therapy [26,80]. Among various 3D printing techniques, FDM coupled with hot melt extrusion (HME) and pressure-assisted syringe-based semi-solid extrusion techniques were used for the fabrication of GRDDSs [25,81,82].

### 4.1. FDM Coupled with HME

FDM combined with HME is currently one of the most studied 3D printing techniques to achieve personalized therapy due to the possibility of complex designs with the inclusion of multiple/single-unit dosage forms, as well as the possibility of combining different APIs, currently one of the revolutionary methods used for tailored medication [83].

HME is the first step and consists of melting the API + polymer/polymer mixture and extruding the molten materials through an extruder barrel, creating non-drug-loaded/drug-loaded filaments. The filaments are then melted in a semiliquid state and extruded through the nozzle of the 3D printer at various temperatures. The extruded material is then applied layer by layer until the designed dosage form is achieved [84]. The most commonly used polymers to obtain dosage forms using this technique are polyvinylpyrrolidone (PVP), polylactic acid (PLA), HPMC, HPC, and polyvinyl alcohol (PVA), among which HPMC, HPC, and PVA are mainly used in order to obtain controlled-release dosage forms [85]. FDM is a flexible method to prepare simple or complex dosage forms with a tailored release. The drug release properties, density, and mechanical resistance of the dosage form depend upon the variation of the percentage of infill (inner layers ensuring the support) and the pattern and number of shells defining the shape of the dosage form. Dosage form size and design play an important role in drug release as various designs can have various surface area to volume ratios; thus, the drug release can vary from one shape to another [86].

**Figure 2:**
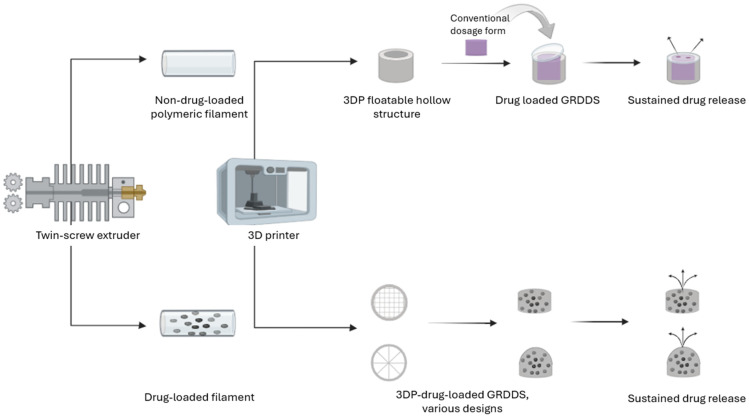
Hollow 3D-printed (3DP) GRDDS enclosing conventional dosage form vs. 3DP-drug-loaded GRDDS.

FDM 3D printing has been exploited for the design of GRDDSs using two strategies. The first one consists of the development of non-drug-loaded polymeric filaments and their subsequent use to create hollow structures through FDM, in which a conventional dosage form is enclosed. The second one relies on the development of drug-loaded filaments, followed by their printing into different designs, with the main aim of creating low-density structures with a sustained drug release [53,87,88]. The two strategies are illustrated in Figure 2. Although it is a very exploited method, it also has some disadvantages compared to PAM, such as the need for high-temperature processing, which makes it almost impossible to use thermolabile drugs/polymers in the formulations [85].

#### 4.1.1. Hollow 3DP GRDDS Enclosing Conventional Dosage Forms

Regarding the first approach, several studies have aimed to design an appropriate hollow structure or shell to obtain the best floating time and to prolong the API release during the floating time. Alqahtani et al. created a dual-compartment dosage form through FDM 3D printing, consisting of an outer non-drug air-filled chamber with no API and an inner compartment in which a propranolol hydrochloride immediate-release tablet was incorporated. The outer layer acts as a sealer, which promotes the gradual release of the API from the inner compartment through a central opening (drug release window) and determines the tablet’s floating capacity. In the study, PVA and PLA filaments (prepared by HME) were used for the 3D printing of the outer shell in order to study the influence of the polymer on the API release time. The results demonstrated that the total floating time (TFT) of the PVA-printed shells was more than 3 h, whereas the PLA-based systems floated for more than 24 h due to the hydrophobic nature of the polymer. Moreover, the smaller the opening size on the outer shell, the longer the floating time and drug release were, while smaller pores reduced the solvent penetration and prolonged the dissolution time [89,90]. Similarly, Jeong et al. developed a novel floating GRDDS using a 3DP capsular device to maintain a sustained release of baclofen in immediate-release tablets. A floating time of more than 24 h was observed, while the average dissolution percentage reached 80% in the 1.7–6.7 h interval [3]. Charoenying et al. produced a floating FDM 3DP shell containing an amoxicillin immediate-release tablet inside to improve patient compliance by reducing the dosing frequency and to obtain a sustained release of amoxicillin from the coated conventional dosage form. PVA was used to print the outer shell, followed by thermal cross-linking. In vitro dissolution studies in a simulated gastric fluid revealed that the 3DP capsular design promoted a longer floating time compared to that of the conventional dosage form. Moreover, the floating time increased after cross-linking. With a longer floating time, a gradual release of amoxicillin was observed, proving that 3DP FDDSs can be used to achieve a sustained release of APIs [91]. The same group proposed another 3D-printed device with a tablet shape to allow the flotation of domperidone tablets (Motilium-M^®^). The device structure consisted of a cap with an internal air chamber to allow floating and a second compartment (the body) with a hole of various diameters, in which the domperidone tablet was placed. The cap was printed using a mixture of hydrophobic PLA and hydrophilic PVA, while the body was made of PLA. PVA was used in the cap as a path to allow water diffusion inside and for the detachment of the air chamber after its complete dissolution, determining the loss of buoyant properties and the elimination of the system from the body. The air chamber ensured anti-flip-up characteristics, and the length of the PVA path governed the TFT, which was up to 24 h in vitro and in vivo for the optimum PVA length. The drug release was controlled by the hole diameter within the body, the optimal value allowing approximately 100% drug release at 24 h [92]. Dumpa et al. created a novel gastroretentive floating pulsatile drug delivery system through HME coupled with FDM. The system enclosed a directly compressed theophylline tablet, and the shell was prepared using HPC-and ethyl cellulose (EC)-based filaments obtained by HME. The study aimed to evaluate the influence of shell thickness and infill density on the floating behavior of the tablets. As a result, the tablets with higher wall thickness and higher infill density were less buoyant. The formulation with a shell thickness of 2 mm and wall thickness of 1.6 mm was selected as the optimum one as it remained buoyant for 6 h [93].

#### 4.1.2. Drug-Loaded 3D-Printed GRDDS

In the second approach, drug-loaded filaments were used to print innovative structures with a reduced density and controlled drug release. Giri et al. formulated a theophylline-containing HPC filament through HME and further obtained intragastric, controlled-release tablets through FDM 3D printing. Similar to previously described studies, the infill density and shell thickness influenced the floating ability and thus, the controlled drug release, but all the developed formulations exhibited a zero-order release kinetics and a 10 h TFT [94]. Lamichane et al. also varied the infill density from 25% to 75% for floating sustained-release cylindrical tablets with pregabalin. The tablets were printed using hypromellose acetate succinate and polyethylene glycol 400 as polymers in two configurations, i.e., a closed system with top and bottom layers and an open system lacking those layers. The study revealed a faster drug release from the open system with a low infill percentage, and an optimized formulation with zero-order drug release, with the partially opened top layer being selected [88]. Mora-Castaño et al. proposed the variation of the internal mesh size of the tablets as another strategy for modifying the density of the product and prolonging the buoyancy. By this approach, the group obtained FDM 3DP gastroretentive systems with a density ranging from 0.73 to 0.82 g/mL, using 10–50% *w*/*w* metformin-loaded HPMC filaments. The effect of the proposed design was evaluated on the floating time as well as on the robustness of the drug release kinetics to assess its suitability for personalized formulations. The proposed approach allowed immediate and prolonged floating and drug release over 8 h, and dose adjustment was possible while keeping the release kinetics unchanged, making the proposed system suitable for personalized therapy [95]. Windolf et al. addressed the need for personalized dosage forms in the treatment of Parkinson’s disease by developing the first printed oral dosage form with a combination of three active substances used to control the symptoms and progress of this disease. In addition to the need for complex drug combinations with various solubilities and absorption profiles, the swallowing difficulties of these patients should be addressed when developing such a system. Thus, the group successfully designed a floating mini-polypill, which was printed using a combination of two filaments: one with pramipexole in PVA for fast release and the other one with a fixed combination of levodopa and benserazide in a prolonged-release matrix of ethylene–vinyl acetate copolymer. To obtain floating polypills and to obtain the desired release profile, the authors tested various geometries as well as the incorporation of various layers and pores in the structure. The mini-polypill design had a reduced density and, consequently, the floating and the in vitro release of pramipexole was fast, while 75% of the API combinations incorporated into the prolonged-release polymer were released within 750 min [96].

In addition to FDDSs, bioadhesive systems are also potential candidates for fabrication using 3D printing. Khizer et al. developed 3DP mucoadhesive tablets using polyethylene oxide (PEO) filaments loaded with gabapentin (prepared by HME) to improve the treatment of an overactive bladder. The study aimed to observe the impact of the infill density and shell number on the drug release rates. Seven matrix tablet formulations were printed, and among all, the formulation with a density of 0.77 mg/mm^3^ (0% infill density and two shells) showed the longest floating time (more than 10 h). The density increased as the infill density and shell number increased, resulting in a decrease in floating time. A decreased floating time was associated with a lower drug release rate and, thus, a lower drug bioavailability. Ex vivo studies showed a high detachment force for all the formulations, as the infill density and shell numbers have no effect on mucoadhesion. In conclusion, gabapentin-loaded 3DP tablets showed a good mucoadhesion and drug release rate; thus, this formulation could be successfully used to achieve a higher drug bioavailability for the treatment of an overactive bladder [42]. To sum up, FDM is currently an intensively studied technique for producing GRDDSs because of its ease and the various strategies that can be applied to achieve the gastroretention of the printed dosage form and sustained release of the active substance.

### 4.2. Pressure-Assisted Microsyringe (PAM) or Semi-Solid Extrusion (SSE)

In addition to HME coupled with FDM 3D printing, some studies used SSE for the fabrication of GRDDSs. SSE 3D printing has the same principle as FDM coupled with HME, i.e., material extrusion, with the main difference being the feedstock materials that are used. While in FDM, the printing material consists of solid filaments, in the case of semi-solid extrusion, a semi-solid material (a mixture of a polymer, solvent, +/− API, and excipient) is used. During the printing process, by applying various temperatures, the semi-solid material is extruded through the printer’s nozzle, and the gel or paste is deposited layer by layer in order to create the desired 3DP dosage form. Using the layer-by-layer printing strategy, a drying process is required after every deposited layer to achieve the desired shape of the dosage form. Compared to FDM, SSE allows lower printing temperatures and higher drug loading, and the use of disposable syringes ensures a lower risk of contamination. However, a disadvantage compared to FDM is the need for a drying process during printing [85,97]. HPMC, PVP, microcrystalline cellulose (MCC), and biopolymers, such as starch, can be used as excipients in these formulations, but HPMC is predominantly used for the development of controlled-release dosage forms [85,98].

Falcone et al. created a ricobendazole-containing drug delivery system (DDS) using SSE. Cross-linked alginate ink was used as a polymer, and HEC was added to thicken the final API suspension. The analysis from the in vitro drug release studies revealed that after 5 h, the formulations sank, but the drug release was extended, reaching almost 24 h [99]. Chen et al. fabricated a clarithromycin floating core-shell system using semi-solid extrusion coupled with FDM 3D printing. The core-shell system consisted of a low-density drug-loaded shell and a floating core that has numerous micro-airbags to promote the buoyancy of the system. In vitro dissolution studies showed no floating lag time, a total floating time of more than 8 h, and a sustained release of 8 h, proving that sustained-release floating dosage forms can be obtained by 3D printing [100]. Thus, semi-solid extrusion is a possible fabrication technique for achieving a sustained release and obtaining various dosage form designs.

## 5. GRDDS Evaluation

### 5.1. In Vitro Evaluation

In vitro methods are the first tests used to study the behavior of a new gastroretentive formulation. Although clinical studies are considered the gold standard for the investigation of GRDDS performance, in vitro tests can offer a good prediction of the gastroretentive behavior in terms of the floating time or drug dissolution rate [101]. In Table 2, we have summarized the most important in vitro methods, classified as general tests, applicable to all GRDDSs, and specific tests used for certain types of GRDDSs. This table highlights the most important general and specific in vitro methods and mentions the parameters monitored during the tests and their applicability for different GRDDSs.

Since most GRDDS formulations are tablets, the most important characteristics that need to be determined through in vitro methods are the following: general appearance, hardness, friability, drug content, uniformity of content, weight variation, and in vitro drug release [5].

In the case of floating DDSs, the key in vitro parameters studied are the floating lag time, total floating time, floating strength, density, porosity, and resultant weight. The floating lag time is defined as the amount of time necessary for the dosage form to emerge on the dissolution medium surface after being placed in it. In the case of effervescent floating tablets, the lag time represents the time required for the reaction between gas-forming agents, like carbonates, and the acidic dissolution medium, which reduces the tablet’s density. Irrespective of the drug, the dissolution medium used is 0.1 N of HCl or a simulated gastric fluid (SGF) in order to mimic the in vivo condition [24,45]. The lack of the ability to mimic the in vitro fed state is a considerable disadvantage as floating DDSs proved to be effective only in the fed state. The lower the floating time is, the lower the chances that the dosage form will be removed from the stomach due to peristaltic waves. The in vitro buoyancy time is defined as the amount of time a system floats during an experiment, being a potential indicator for in vivo gastric retention time, and similar to dissolution tests, it is measured in 0.1 N of HCl solution. The GRDDSs produced by conventional manufacturing techniques are buoyant either through effervescence or due to the properties of the materials used in their preparation, i.e., low density, raft-forming capacity, or gelling capacity. For the former ones, the buoyancy is lost over time due to polymer erosion or dissolution; so, the performance of the product mainly depends on the formulation. On the contrary, for 3D-printed structures, the floating performance, reflected by a reduced floating lag time and increased floating time, can be easily modulated through both formulation and design considerations. The use of printed non-drug-loaded devices incorporating conventional dosage forms allows the modulation of flotation time through the incorporation of air pockets within the structure, with this approach resulting in a floating time of 24 h or higher [3]. If the printed floating dosage form incorporates the API in its structure, the floating time is generally related to the density of the product, modulated through changes in the infill, number of printed layers, shell thickness, or incorporation of pores in the structures. When using these strategies, the maximum reported floating time was variable (6–14 h). While most of the studies showed a dependency of the total floating time on the density of the product, Lamichane et al. observed that the floating time was mostly influenced by the presence of top/bottom insulation layers, which could prevent water entrance in the system and ensure buoyancy over 24 h [88]. Khizer et al. used shell number and infill density to change the floating behavior and observed a decrease in floating time when density increased, with the best floating ability (floating time over 10 h) being reached at a density of 0.77 mg/mm^3^ (2 shells and 0% infill). They also found that both the density of the product and the number of outer layers were important since the formulation with a single outer layer (although it had a lower density) had a shorter floating time [42]. Even though an infill of 0% favors flotation, this value can make it difficult to print the product, as found by Chen et al., who proposed the use of an infill between 15 and 25% for easy printing, proper flotation, and good mechanical resistance of the print [109]. In vitro dissolution testing can be problematic for buoyant dosage forms as the contact surface between the dosage form and the dissolution medium is very low. A possible method to impede flotation is to use helical wire sinkers during dissolution testing [24,89]. To obtain a sustained release from the printed structures, various design strategies have been reported. When conventional tablets are incorporated in 3D printed capsular/tablet-shape devices, the API release from the internal dosage form is controlled by the outer shell design, i.e., the presence of pores and their size and number, as well as the interaction between the polymer and the dissolution medium. The presence of pores/holes in the outer shell provides the entrance path for water in the drug reservoir and a window for drug release. Thus, when a single whole is present in the outer shell, the drug release rate increases with its diameter. Alqahtani et al. reported a higher drug release when the diameter of the whole increased from 1 to 4 mm, as well as a change in the release kinetics of propranolol from the zero order (pore diameter of 1–2 mm) to the first order (pore diameter of 3–4 mm). In addition, the interaction with the dissolution medium is governed by the hydrophilic/hydrophobic nature of the shell and the cross-linking degree of the main polymer. Alqahtani et al. observed that a floating device made of PLA will exhibit a more sustained release than PVA for propranolol from an immediate-release tablet that is filled inside (10 h vs. 2 h) due to differences in their affinity for the simulated gastric fluid [89]. To achieve a sustained release using a PVA device, Charoenying et al. proposed the thermal cross-linking of the polymer and observed that the higher the cross-linking temperature and time, the greater the degree of cross-linking and the lower the water uptake; so, these processing parameters may be optimized to modulate the drug release. For the printed dosage forms that embed APIs in the filament used for printing, the nature of the filament-forming polymer and drug loading of the filament have a positive influence on the dissolution rate [92]. In this regard, Mora-Castaño et al. showed that the drug released after 8 h from 3D-printed gastroretentive systems increased from 70% from printed systems containing 10% and 20% APIs to 90% for filaments with 30–40% APIs [95]. In terms of polymers, Windolf et al. designed a sustained-release floating mini-polypill by incorporating one API in a PVA-based rapid drug release layer and combined APIs in an ethylene–vinyl acetate copolymer matrix for a prolonged drug release (75% after 750 min) [96].

Density evaluation is crucial for FDDSs as it determines floatability. In the case of single-unit dosage forms, the weight and volume of the tablet should be measured. However, in multiple-unit systems such as microspheres, density can be calculated either by determining the volume of the known mass of microspheres, by the photographic counting method, or by the liquid displacement method, using water, benzene, or n-Hexane as solvents. For 3D-printed dosage forms, density is proportional to the infill density and shell number [42]. A study by Mora-Castaño et al. showed a variation in density from 0.73 to 0.82 g/mL with the change in the internal mesh size of the tablets [95].

Porosity is another key parameter for FDDS evaluation, and it can be determined based on an equation that uses the true density and particle density of the system [110]. True density can be measured by a helium-air pycnometer, nitrogen-adsorption method, or mercury porosimetry. The resultant weight indicates the polymer’s hydration and erosion capacity and can be measured by using a resultant weight apparatus, as described by Parikh et al. [47]. The water uptake rate of the matrix is directly proportional to the release rate of the drug; thus, it is an important parameter to evaluate.

In the case of raft-forming systems, the most important indicators for in vivo performance are the raft (gel) strength, sol-gel transition temperature and gelling time, polymer’s water uptake capacity, buoyancy time, viscosity and rheological properties, and resistance to gastric reflux, of which, only the first three parameters can be verified in vitro. The raft strength measures the force (expressed in g) required to pull an L-shaped stainless steel wire probe up through the raft. The results are recorded using a texture analyzer and reflect the gelling capacity of the prepared formulations [37,111]. The sol-gel transition temperature is defined as the temperature at which the phase transition is observed when the formulation is kept in a sample tube and heated at a specific rate. The gelling time represents the time required for the formation of the raft and solely depends on the quantity of the gel-forming polymer [111]. The water uptake capacity of the polymer is another specific parameter that is determined using Equation (1), as shown below:

(1) % Water uptake=((W2−W1)/W2)×100

where W1 represents the initial weight of the formed gel when the polymer is placed in a solution of 0.1 N of HCl at 37 ± 0.5 °C and W2 is the weight of the gel after a 30-min excess-water decanting interval [35]. The viscosity and rheological properties of raft-forming systems are important parameters that can predict a possible difficulty during the administration of the drug by the patient and are thus important for ensuring good patient compliance and safety [111]. Resistance to gastric reflux is one of the specific parameters that indicate the maximum force required to break the raft through a 10-mm orifice, simulating the size of the esophageal sphincter, with a 9-mm cylindrical rod fitted to the arm [37].

The swelling index, water absorption rate, and exposed size are key parameters to investigate for the successful development of an expandable system. The swelling index or water uptake capacity can be determined by using Equation (2), which takes into consideration the weight of the dry tablet (Wd) and the weight of the swollen tablet (Ws) in the dissolution medium [5,41,107]

(2) Q=((Ws−Wd)/WD)×100

Several methods were designed to determine the water absorption rate, taking into consideration the possible system degradation as the sample needs to be removed from the dissolution medium and weighed at predetermined times.

The exposed size needs to be evaluated for unfolding expandable systems in order to determine their maximum expansion. This parameter is evaluated by the measurement of the dosage form’s unfolding behavior at various time intervals [5,41,107].

In conclusion, several in vitro tests (general and specific ones) can be applied to ensure that the formulation meets the expectations and that GRDDSs are successfully formulated.

### 5.2. In Vivo Tests

Novel in vivo imaging techniques, such as Magnetic Resonance Imaging (MRI), scintigraphy, or X-ray allow the visualization and tracking of the dosage form in vivo in a non-invasive way [5,101]. There are many other imaging methods, such as computed tomography, positron emission tomography, and single-photon emission computed tomography, but these methods were not used for the evaluation of in vivo properties of the dosage forms [112]. The results from in vitro methods will probably differ from in vivo tests based on the complexity of the gastrointestinal tract. Thus, both the pH and the viscosity will constantly change based on various factors, like the amount or quality of food, and these changes will have a significant impact on the behavior of GRDDSs. There is a need for in vivo studies, either in animal models or in humans, to attest to the efficacy of GRDDSs. First, in vivo tests are usually carried out in animal species, like rats or rabbits. However, there are major differences in the stomach physiology of humans and animals, such as different gastric transit times, which can lead to different results. Then, the dosage forms designed for human use are administered to pigs, rabbits, or dogs [79,101].

Gamma scintigraphy is a useful method for determining the gastroretention of the dosage form in humans, and it relies on the incorporation of a small amount of radioisotope with a short half-life within the dosage form. Then, the formulation is exposed to a neutron source to release the characteristic gamma rays that will be captured on a computer as an image that needs to be processed [5,113]. Scintigraphy is the gold standard of dosage form tracking because of a high temporal resolution and sufficient spatial resolution [54]. Gansbecke et al. successfully analyzed the gastroretentivity of a floating dosage form in 1991 using scintigraphy by administering a meal containing a radionuclide with a short half-life, i.e., 99 mTc, in order to reveal the contour of the stomach, and a second radionuclide with a longer half-life was used to label the dosage form [114]. Ali et al. (2007) carried out in vivo studies with gamma scintigraphy, using rabbits as models, to assess the buoyancy and pharmacokinetic parameters of a dosage form containing metformin HCl. A comparative pharmacokinetic study was also conducted by administering optimized HBS capsules and immediate-release capsules, both with radiolabeled metformin, using SnCl2 as a radiolabeling agent [52]. Although this method seems to be efficient, it also has drawbacks as in this method, there is a lack of anatomical information; so, it may be unclear whether the formulation is located in the stomach or in the nearby segment of the duodenum [101].

Radiology or X-ray is an alternative to evaluate the gastroretentivity and disintegration rate of dosage forms, using a radio-opaque material in the composition of the dosage form. Ahmed et al. analyzed itopride hydrochloride expanding tablets using X-ray in two healthy male volunteers in fasted and fed states. X-ray was used to observe the position, behavior, and residence time of expanding tablets in the stomach, using barium sulfate as a radio-opaque material replacing the API. X-ray provided detailed images, such as the difference between the fed and unfed state [40]. Rahamathulla et al. analyzed the GRT of losartan potassium effervescent floating matrix tablets combined with a radio-opaque material through X-ray imaging using albino rabbits. The transition of the dosage form through the GIT was easily observed using the proposed method [115]. Although an effective method, similar to scintigraphy, X-ray has the same disadvantages as scintigraphy, mainly the radiation exposure to the volunteers and the missing anatomical references [101].

MRI is another technique that is used for studying the in vivo behavior of GRDDSs, and it is one of the best techniques that provides an anatomical reference with sufficient spatial resolution [101]. A great advantage of this in vivo technique is that the radiation exposure is significantly reduced as this safe method uses magnetic fields and radio waves to observe complete anatomical structures [5]. To use this method, a compound with superparamagnetic properties (e.g., ferrous oxide) is included in the dosage form. A magnetic dipole signal and appropriate sensors are used to visualize the dosage form in vivo in 3D. Considering that in this method, extremely sensitive sensors are used, it also requires a magnetically shielded room to reduce the noise of the Earth’s magnetic field [116,117]. Curley and colleagues demonstrated that the in vivo disintegration of oral paracetamol formulations could be easily visualized using this technique [118]. Marciani et al. successfully used MRI to study the gel properties of an alginate gel matrix raft-forming system containing CO_2_ in healthy volunteers and concluded that echo-planar magnetic resonance imaging shows great potential in assessing the in vivo raft formation of alginates [119]. Furthermore, methods that are less used in vivo tests include gastroscopy, ultrasonography, and a 13C octanoic acid breath test. Gastroscopy is a per-oral endoscopy, which requires the use of optical fibers and a video camera to determine the location of a GRDDS. Although it is a precise technique, it is less convenient as it can require minor anesthesia. Thus, it is rarely used for the evaluation of GRDDSs [1,5]. Ultrasonography uses ultrasounds to view the internal body structures and generates ultrasonic waves to produce images of the abdominal organs. The method allows the determination of the GRT and the intragastric location of the GRDDS, the evaluation of the solvent penetration into the gel, and the interactions between the dosage form and the gastric mucosa during peristalsis [1,6].

The ^13^C octanoic acid breath test assesses the extent of the absorption of the drugs from GRDDSs using radioactive ^13^C octanoic acid [6]. It is an easy and inexpensive method to determine gastric emptying and the impact of food on gastric motility; so, it could be a useful method for further studying the impact of food over the gastric residence time of GRDDSs in the fed/unfed state, but further information is needed [120].

## 6. GRDDSs on the Market

As shown in Table 3, several GRDDSs on the market are aimed at addressing Parkinson’s disease treatment, which requires the co-administration of at least two active substances, with levodopa and benserazide being frequently combined. This therapeutic strategy and the need to saturate the receptors in the upper gastrointestinal tract with levodopa justifies the interest in the development of floatable systems with the combination of levodopa and benserazide/benserazide hydrochloride. Additionally, the need for individual dosages and adjusted drug release during the treatment, as well as the need to combine these two APIs with a third one, for example, pramipexole, as the disease progresses makes 3D printing an attractive manufacturing method that is able to provide dosage forms with multiple APIs, with individually adjusted-release profiles and adequate gastric residence times. In this regard, Windolf H et al. successfully designed floating tablets and mini-tablets combining levodopa, benserazide, and pramipexole, which allow a facile dose adjustment during the treatment, in accordance with the needs of the patients, without changing the release profile of the APIs while facilitating swallowing at the same time [96].

GRDDSs for active substances with a narrow absorption window, such as metformin hydrochloride, are also relevant both as products on the market and as 3D-printed GRDDSs. Despite its thermostability, which makes the processing at high temperatures possible, which is required for HME and FDM, this API is challenging for 3D printing due to the high therapeutic doses, which are used as 500 mg or 1000 mg tablets. Three-dimensional printing allowed the preparation of floatable tablets, using up to 40% metformin-loaded cellulose-based filaments, in which the dose of the API can be adjusted according to patients’ needs by changing the internal mesh size while preserving the release kinetics and floating ability [95].

Several antibiotics are found on the market as GRDDSs, generally due to their better absorption in the upper gastrointestinal tract, instability at a high pH value, or their use in the treatment of *H. Pylori* infection. Several marketed products are available with ciprofloxacin, ofloxacin, and cefaclor. Regarding the proposed 3D printing-based strategies for the gastroretention of antibiotics, Charoenying et al. proposed a floating 3D-printed device that can embed conventional commercial dosage forms, including capsules, tablets, powders, and caplets. This way, gastroretention can be achieved for any antibiotic or other APIs of interest, and the high dose of the active substance will not represent an impediment as long as the floating device is devoid of the active substance [92].

## 7. Future, Challenges, and Expected Impact of 3DP GRDDSs

The interest in 3D printing has grown exponentially in many fields since its origin, encouraging the development of applications in the pharmaceutical domain. It is expected that in the future, the advantages of this technology will be used to develop GRDDSs that are more complex and better adapted to their purpose. Also, more 3D printing techniques will be applied in the manufacture of GRDDSs.

In the future, this technology will probably have the most important impact in the manufacture of personalized GRDDSs, considering that traditional manufacturing methods are not adapted for this purpose. This will enable a tailored API release based on every patient’s need, with 3D printing being able to overcome a major challenge, i.e., to attain flexibility regarding the API dose and drug release rate. Three-dimensional printing is one of the best candidates for producing tailored medication due to the variable production methods and the variability of dosage form designs, which can lead to differences regarding the drug release rate [126,127].

For large-scale manufacturing, the quality, stability, and safety of 3DP GRDDSs will have to be considered. Regarding quality, there are no specific standards for 3DP dosage forms, with the requirements for conventional dosage forms being adapted for the printed dosage forms too. Furthermore, stability and safety issues are rarely addressed by current studies. In addition, this technology is more difficult to scale up compared to conventional manufacturing technologies. On the other hand, the manufacture of personalized dosage forms in community or hospital pharmacies has recently been proposed. This objective will be possible in the future by adapting logistics, suitable personnel training, and potentially, cooperation with industry or research groups [128]. However, this requires the rapid development of the formulation and manufacturing conditions, which will be facilitated through the intervention of artificial intelligence (AI) [129]. Due to the advancements in these two fields, i.e., 3D manufacturing and AI, we can now foresee that 3D technology will have a significant impact on the manufacturing of patient-specific GRDDSs in the future.

## 8. Regulatory Considerations

3D printing is a technology that has recently made its way into the market and is currently considered an alternative, advanced manufacturing technology. To date, there have been no recommendations or guidelines for the use of 3D printing in pharmaceutical production issued by regulatory authorities worldwide; so, the regulatory approval of 3DP drug products may be a barrier for their development.

From the regulatory perspective, there is a distinction between compounded and manufactured medicines. Personalized 3DP products can be considered as compounded extemporaneously if they are prepared for an individual patient or tailored for an identified need [130]. In this regard, the destructive quality testing methods applied at the end of the manufacturing process for the drug products are not applicable to 3D-printed personalized medicines since they are produced in small amounts or on demand. A proposed solution for this problem is the use of Process Analytical Technology (PAT) tools, such as near-infrared and Raman spectroscopy, which have been shown to be adequate non-destructive tools for the quality control of 3DP drugs [131]. Regarding the licensed products, in terms of regulations, they can fall either under the incidence of the regulations for medical devices or for medicinal products, with different requirements for being on the market. Some regulatory considerations for 3DP medical devices were proposed in 2017 by the FDA in a draft guidance entitled “Technical Considerations for Additive Manufacturing of Medical Devices”. According to this, specific regulations are needed for each 3D printing method due to their particularities. The guideline brings to light the most stringent problems to be regulated for the products manufactured through this technology, i.e., quality control, process validation, hardware and software requirements, and testing of the equipment, but the regulations for medicinal products are still missing, although the quality criteria are generally stricter for these than for medical devices [132]. In the European Union, only the manufacturers of 3D printers used for the fabrication of medical devices and drugs have a legal framework to establish the necessity of a risk assessment to evaluate the health and safety requirements applicable to the use of the equipment [133].

The regulatory concerns for the approval of 3DP GRDDSs are related to the quality requirements that the final product should meet, the quality of the materials used, the consistency and reproducibility of the printing process, and the availability of 3D printers for pharmaceuticals that meet the GMP requirements [130,134,135]. In terms of quality, it is proposed that real-time quality assurance using tracking measures represents the foundation of product quality and safety instead of quality control, with this strategy being able to promote the application of 3D printing in clinical practice [130]. However, appropriate quality standards and performance requirements must be established by the regulatory bodies to ensure the successful clinical application of 3D-printed products.

## 9. Conclusions and Expert Opinion

GRDDSs show promising results for the future development of dosage forms using drugs with a narrow absorption window, instability at an alkaline pH, and high solubility and absorption at an acidic pH. As these systems offer a prolonged gastric residence time and an extended release of the API, they have the potential to increase patient compliance and address local disease therapy. Moreover, in recent years, new emerging technologies have been proposed to expand the existing manufacturing techniques of GRDDSs.

One of those is 3D printing, which brings high flexibility in terms of API concentration, multiple APIs in the same dosage forms, and tailored drug release, which allows the development of complex GRDDSs according to the requirements of personalized therapy. Using 3D printing in the manufacturing of GRDDSs on a large scale is dependent on the standards for quality, efficacy, safety, and regulatory aspects. From the regulatory perspective, for personalized medicine, the preparation of GRDDSs as magistral compounding fits better in comparison to their release as registered medicinal products.

Regarding quality, considering there are no specific standards for 3DP dosage forms, theoretical general tests and specific tests that are applicable to GRDDSs may be used. For magistral compounding, generally produced in small amounts, applying destructive quality control methods can be difficult, and non-destructive PAT tools such as near-infrared and Raman spectroscopy can be an alternative.

Applying 3D printing for the manufacturing of magistral compounding requires the rapid development of the formulation and manufacturing conditions. This may be achieved through the intervention of AI within the formulation-by-design paradigm.

## Figures and Tables

**Table 1:** Preparation techniques and dosage forms for GRDDSs.

GRDDS	Dosage Forms and Preparation Techniques	References
Single-unit floating system	Layered pellets obtained in a fluidized bed processor Tablets obtained by wet granulation Tablets obtained by direct compression Tablets or capsular devices fabricated by HME/semi-solid extrusion coupled with FDM In situ gelling liquid formulation	[23,24,25,26,27,28,29,30]
Multiple-unit floating system	Granules with APIs obtained by wet granulation Foaming encapsulated particles prepared by ultrasonic batch technology Mini-tablets with encapsulated microparticles, prepared by direct compression	[23,31,32,33,34]
Raft-forming system	Tablets obtained by solid dispersion preparation using solvent evaporation method	[35,36,37,38,39]
Magnetic system	Press-coated tablets with internal magnets	[2,21]
Expandable system	Tablets prepared by direct compression	[1,40,41]
Mucoadhesive system	Directly compressed tablets with multiple-unit microparticles prepared through emulsification–internal gelation method	[1,42,43,44]

**Table 2:** In vitro tests used to evaluate the properties of the gastroretentive formulations.

Applicability	Parameters	Methods	References
**General In Vitro Tests**			
All GRDDSs	Drug release rate and kinetics	Dissolution test (0.1 N HCl as dissolution medium)	[5,101]
	Surface morphology	Scanning electron microscopy (SEM)	[5]
	Stability studies	Stability prediction algorithm (physical appearance, drug content, and drug release profile)	[23,102]
	Porosity and bulk density of dosage forms	Densitometry	[63,101,103]
	Drug and excipient physical and chemical interaction studies	Near-infrared spectroscopy (NIR), HPLC, DSC, and IR spectroscopy	[28,38,56,58,104]
Specific tests			
Mucoadhesive systems Multiple-unit systems containing microspheres	Drug entrapment efficiency	HPLC	[43,105]
FDDSs, expandable systems, and raft-forming systems	Floating lag time (FLT), total floating time, floating strength	Dissolution test	[23,46,63]
	Swelling index (SI)	Dosage form submersion in test medium, removed at predefined time and weighed	[63,103,106,107]
Ion-exchange resin systems	Binding efficiency	Binding assay	[76]
Foaming FDDSs	Foam density	Densitometry	[33,35]
	Foam structure	Microtomography system using 2D/3D analysis	[33,35]
Raft-forming systems	Raft strength and raft volume	Physical balance	[38,39,40]
Mucoadhesive systems	Tensile strength and detachment force	Diametrical compression test	[108]
FDDSs, swelling systems, and mucoadhesive systems	Gel strength	Texture analysis	[20,76,106]

**Table 3:** Gastroretentive products available on the global market [5,21,47,121,122,123,124,125].

Active Substance	Technology	Brand Name	Manufacturer
Prazosin HCl	Effervescence- and swelling-based floating system	Prazopress XL^®^	Sun Pharma, Gujarat, India
Carvedilol	Osmotic system	Coreg^®^	Glaxosmithkline, Philadelphia, PA, USA
Verapamil HCl	OROS	Covera HS^®^	DURECT Corporation, Cupertino, CA, USA
Nisoldipine	Geomatrix^TM^	Sular^®^	Skyepharma, Shionogi Pharma Inc., London, UK
Misoprostol	Bilayer floating capsule	Cytotec^®^	Pfizer, Sandwich, UK
Levodopa and benserazide	HBS floating capsule	Madopar HBS^®^	Roche, Hertfordshire, UK
Levodopa and benserazide hydrochloride	HBS floating capsule	Prolopa HBS^®^	Roche, Hertfordshire, UK
Diazepam	HBS floating capsule	Valrelease^®^	Roche, Hertfordshire, UK
Simethicone and aluminum–magnesium salts	Floating and swelling systems	Inon Ace Tablets^®^	Sato Pharma, Akasaka, Japan
Metformin HCl	Minextab Floating^®^: floating and swelling systems	Metformin HCl	Galanix, Pessac, France
Cefaclor LP	Minextab Floating^®^: floating and swelling systems	Cefaclor LP	Galanix, Pessac, France
Tramadol LP	Minextab Floating^®^: floating and swelling systems	Tramadol LP	Galanix, Pessac, France
Ofloxacin	Effervescent floating system (film-coated tablet)	Zanocid OD^®^	Ranbaxy, Mumbai, India
Metformin hydrochloride	Effervescent floating system (film-coated tablet)	Riomet OD^®^	Ranbaxy, Mumbai, India
Ciprofloxacin	Effervescent floating system (film-coated tablet)	Cifran OD^®^	Ranbaxy, Mumbai, India
Ferrous sulfate	Colloidal gel-forming floating system	Conviron^®^	Ranbaxy, Mumbai, India
Ciprofloxacin hydrochloride	Erodible matrix-based system	Cipro XR^®^	Bayer, Whippany, NJ, USA
Carbidopa/levodopa	Expandable system (unfolding)	Accodrion Pill^®^	Intec Pharma, Tel-Aviv, Israel
Aluminum–magnesium (antacids)	Raft-forming system	Topalkan^®^	Pierre Fabre Medicament, Paris,, France
Ciprofloxacin	Polymer-based swelling system: Acuform^TM^	proQuin XR^®^	Depomed, Newark, CA, USA
Metformin hydrochloride	Polymer-based swelling system: Acuform^TM^	Glumetza^®^	Depomed, Newark, CA, USA
Metformin hydrochloride	Polymer-based swelling system: Acuform^TM^	Metformin GR^TM®^	Depomed, Newark, CA, USA
